# Supplementary figures and images for: In silico Prioritization of Transporter–Drug Relationships From Drug Sensitivity Screens
Source: Front Pharmacol. 2018 Sep 7;9:1011. doi: 10.3389/fphar.2018.01011 (PMC6137680; doi:10.3389/fphar.2018.01011)

**Supplemental Figure 1.** PubMed search of drug gene associations.

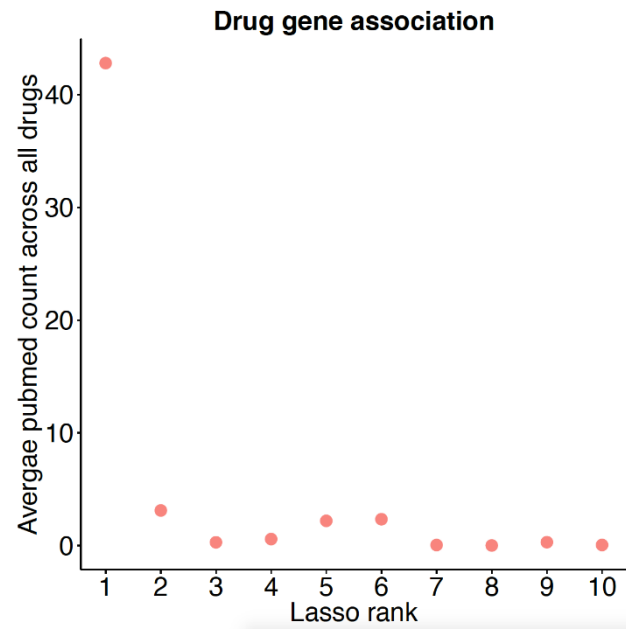

Supplement: FIGURE S1 — PubMed search of drug gene associations. [file Image_1.pdf]
